# Rapid Subcutaneous Migration of *Dirofilaria repens* Nematode in Facial Tissue, Italy

**DOI:** 10.3201/eid3106.241915

**Published:** 2025-06

**Authors:** Mariaelisa Carbonara, Simona Gabrielli, Alessia Ricci, Roberta Iatta, Riccardo Paolo Lia, Maria Virginia Tomassi, Andrea Mariano, Jairo Alfonso Mendoza-Roldan, Domenico Otranto

**Affiliations:** University of Bari Aldo Moro, Bari, Italy (M. Carbonara, A. Ricci, R. Iatta, R.P. Lia, J.A. Mendoza-Roldan, D. Otranto); University of Rome Sapienza, Rome, Italy (S. Gabrielli); National Institute for Infectious Diseases Lazzaro Spallanzani, Rome (M.V. Tomassi, A. Mariano); City University of Hong Kong, Kowloon, Hong Kong, China (D. Otranto)

**Keywords:** vector-borne infections, zoonoses, human dirofilariosis, parasites, *Dirofilaria repens*, diagnosis, Italy

## Abstract

We report a *Dirofilaria repens* nematode infection in a woman in Italy who sought care for a fast-creeping lesion within her subcutaneous facial tissue. Dirofilariosis should be included in differential diagnosis of subcutaneous nodules or creeping lesions. This case highlights the need for controlling canine dirofilarioses to mitigate zoonotic risk.

Dirofilarioses are mosquitoborne zoonotic diseases caused by filarial nematodes, which affect domestic and wild carnivores in tropical, subtropical, and temperate areas worldwide (*1*). Humans act as dead-end hosts because the third stage larvae, which are transmitted by mosquitoes during the blood meal, do not usually reach sexual maturity. Human cases of dirofilariosis have been documented worldwide; *Dirofilaria immitis* and *D. repens* nematodes have been reported from both the New and Old World (*2*). In humans, subcutaneous lesions caused by *D. repens* nematode infection can occur in several anatomic areas (e.g., forehead, arms, and periorbital and perioral areas) and, rarely, in deeper tissues (e.g., lymph nodes, lungs, muscles, and dura) (*3*). In addition, pulmonary localization of *Dirofilaria* spp. is characterized by the presence of solitary, well-circumscribed, noncalcified peripheral subpleural pulmonary nodules (coin lesions), which mimic lung cancer (*4*). In the Mediterranean Basin, ideal climatic conditions for the development of mosquito vectors, as well as the high prevalence of microfilaremic dogs, are risk factors for human infections, as observed in areas highly endemic for canine dirofilariosis, such as southern Italy (*5*,*6*). Specifically, in Europe, *D. repens* is considered an emerging pathogen, because it presents an expanding distribution linked to an increasing number of human cases (*4*). We report a case of human dirofilariosis in a woman in her forties, living in Rome, Italy, with 3 cats as pets.

The patient first underwent ophthalmologic consultation because of visual impairment; her condition was initially misdiagnosed as an allergic reaction of the right upper eyelid. Five days later, the patient was referred to the National Institute for Infectious Diseases Lazzaro Spallanzani after she reported a worm-like organism creeping within the subcutaneous tissue of the right lower periorbital region (Video; Appendix Figure). No other clinical signs (e.g., dermo-epidermal eruptive patches) were recorded besides retroauricular lymphadenomegaly and low-grade fever (up to 38.0°C). The patient had not recently traveled abroad. 

**Video:** Video showing lesion caused by fast-migrating *Dirofilaria repens* nematode in the facial tissue of a woman from Rome, Italy.

After signing informed consent, the patient was hospitalized but surgery was not performed because the suspected parasite (likely *Dirofilaria* spp.) had migrated to the right parietal area of the head (i.e., the subcutaneous tissue at the parietal bone of the skull), preventing its removal. During her 5-day hospitalization, the patient was in good clinical condition; hematological and serologic biochemical parameters were within reference ranges; eosinophilia was not present. Infections by *Strongyloides stercoralis* and zoonotic filarial worms (i.e., *Brugia* spp., *Wuchereria bancrofti*, *Mansonella* spp., and *Oncocherca* spp.) were excluded by serologic assays (i.e., commercial ELISA kits). Chest radiography was performed to exclude the presence of coin lesions typical of *Dirofilaria* spp. infection. 

We tested a serum sample at the Department of Public Health and Infectious Diseases Sapienza, University of Rome, to assess exposure to *Dirofilaria* spp. by using an in-house ELISA based on somatic antigens of adult *D. repens* (*6*,*7*), which yielded positive results (i.e., optical density 1.56; optical density cut off 1.03 for *D. repens*). The woman was discharged from the hospital with the recommendation to return on observation of parasite reemergence to the facial subcutaneous tissue. 

Two weeks later, the nematode migrated in the frontal area, and a surgical excision was performed under local anesthesia. The specimen was shipped to the University of Bari (Italy) for further morphological and molecular analysis. The fragmented nematode was morphologically identified as a mature female, cylindrical, ≈2.96 cm in length, and 0.480 mm thick (Figure 1). Microscopic analysis revealed a thick laminated cuticle with characteristic longitudinal ridges and cross-striations (Figure 1), leading to the identification of the parasite as *D. repens* (*8*). 

**Figure 1:**
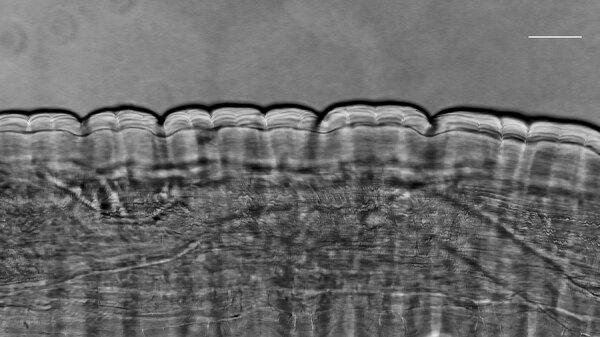
Microscopic view of *Dirofilaria repens* nematode extracted from subcutaneous facial tissue of a patient, Italy. Microscopic analysis revealed a thick laminated cuticle with characteristic longitudinal ridges and cross-striations, leading to the identification of the parasite. Scale bar indicates 100 µm.

Genomic DNA was extracted from the nematode and tested by conventional PCR targeting *cox*1 gene (*9*) to obtain a reference sequence. BLAST (https://blast.ncbi.nlm.nih.gov) analysis revealed 100% nucleotide identity with reference sequence of *D. repens* in the GenBank database (accession no. MW675692), which further confirmed by phylogenetic analyses (Figure 2). At the 5-month follow-up, the patient’s only residual symptom was a persisting uncomfortable feeling, likely associated with parasite migration. We also performed specific tests to detect *Dirofilaria* spp. on her pets, yielding negative results.

**Figure 2:**
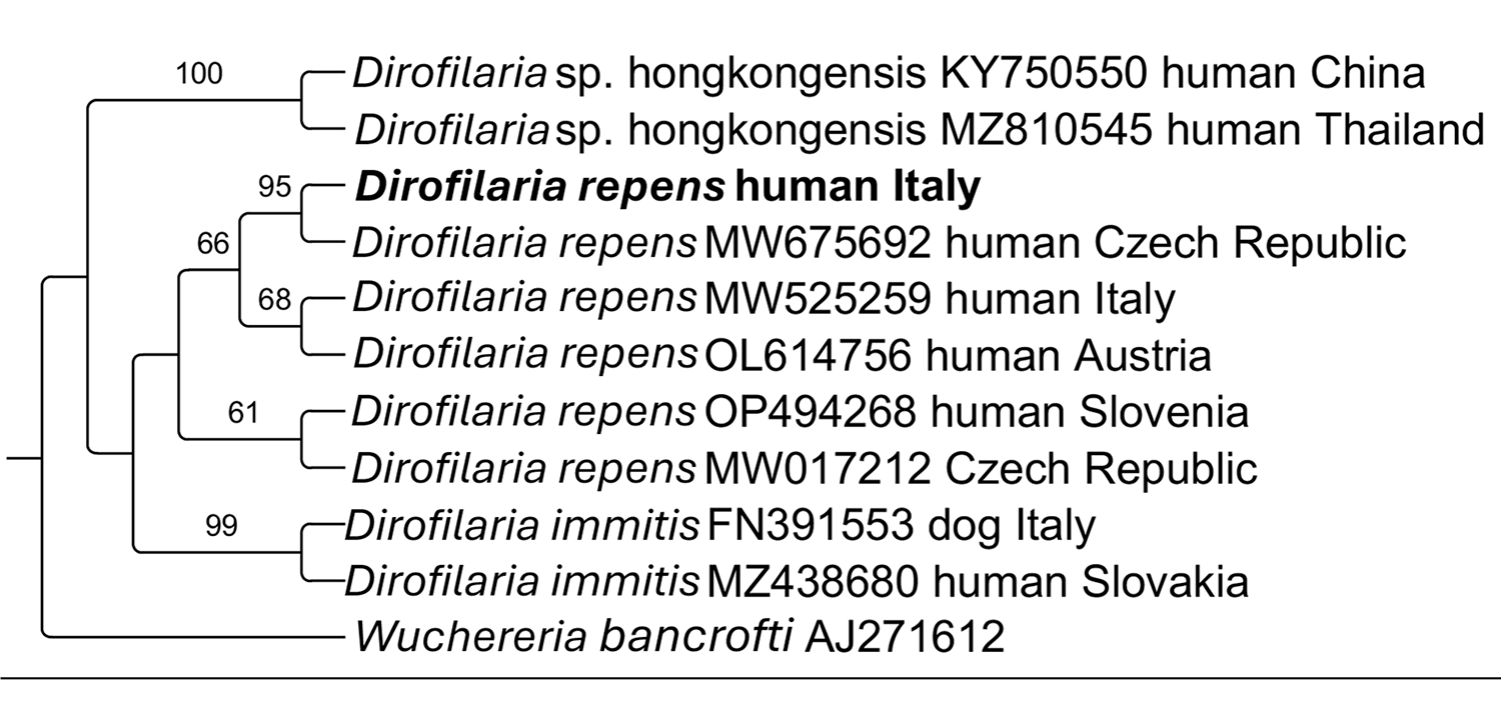
Phylogeny of *Dirofilaria repens* based on *cox*1 gene sequences in study of rapid subcutaneous migration of *D. repens* nematode in facial tissue, Italy. The sequence from this study is shown in bold. *Wuchereria bancrofti* was used as outgroup. Bootstrap confidence values (1,000 replicates) are shown at the nodes only for values >60%.

The increasing incidence of human cases of dirofilariosis in Europe (*10*) underscores the need for including this emerging zoonotic disease in the differential diagnosis of pulmonary or subcutaneous nodules in absence of eosinophilia. The rapid migration of the nematode in this case was unusual, highlighting the variability of clinical signs in patients infected by *D. repens* nematodes, which range from stationary nodules to fast-migrating lesions in subcutaneous tissues. Human dirofilariosis is typically an abortive infection because humans are accidental hosts, and microfilaremia is absent (*1*). Consequently, traditional diagnostic methods applied in veterinary medicine (e.g., Knott’s test) are unsuitable. Definitive diagnosis in human patients is challenging, and often only achievable after surgical removal of the parasite. 

In summary, we identified *D. repens* nematode infection in a woman with a creeping lesion in her subcutaneous facial tissue. This case highlights the need for a One Health approach in implementing vector control strategies and regular monitoring of reservoir hosts in endemic areas to mitigate the risk for human *D. repens* infection.

Appendix Additional information about rapid subcutaneous migration of *Dirofilaria repens* nematode in facial tissue, Italy
