# Supplementary figures and images for: Rapid Subcutaneous Migration of Dirofilaria repens Nematode in Facial Tissue, Italy
Source: Emerg Infect Dis. 2025 Jun;31(6):1258–60. doi: 10.3201/eid3106.241915 (PMC12123934; doi:10.3201/eid3106.241915)

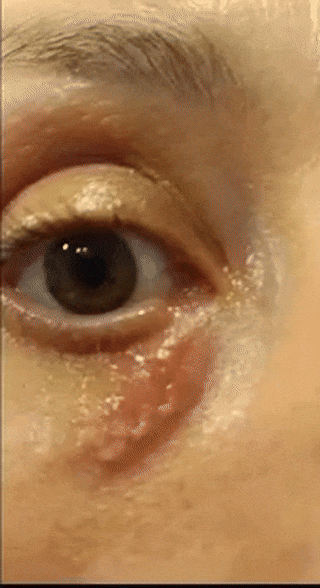

Supplement: Supplementary file 1 [file 24-1915-V.gif]
