# Supplementary material for: Rapid Subcutaneous Migration of Dirofilaria repens Nematode in Facial Tissue, Italy
Source: Emerg Infect Dis. 2025 Jun;31(6):1258–60. doi: 10.3201/eid3106.241915 (PMC12123934; doi:10.3201/eid3106.241915)
Supplement: Appendix — Additional information about rapid subcutaneous migration of Dirofilaria repens nematode in facial tissue, Italy [file 24-1915-Techapp-s1.pdf]

Article DOI: https://doi.org/10.3201/eid3106.241915

EID cannot ensure accessibility for supplementary materials supplied by authors. Readers who have difficulty accessing supplementary content should contact the authors for assistance.

Rapid Subcutaneous Migration of *Dirofilaria repens* Nematode in Facial Tissue, Italy

Appendix

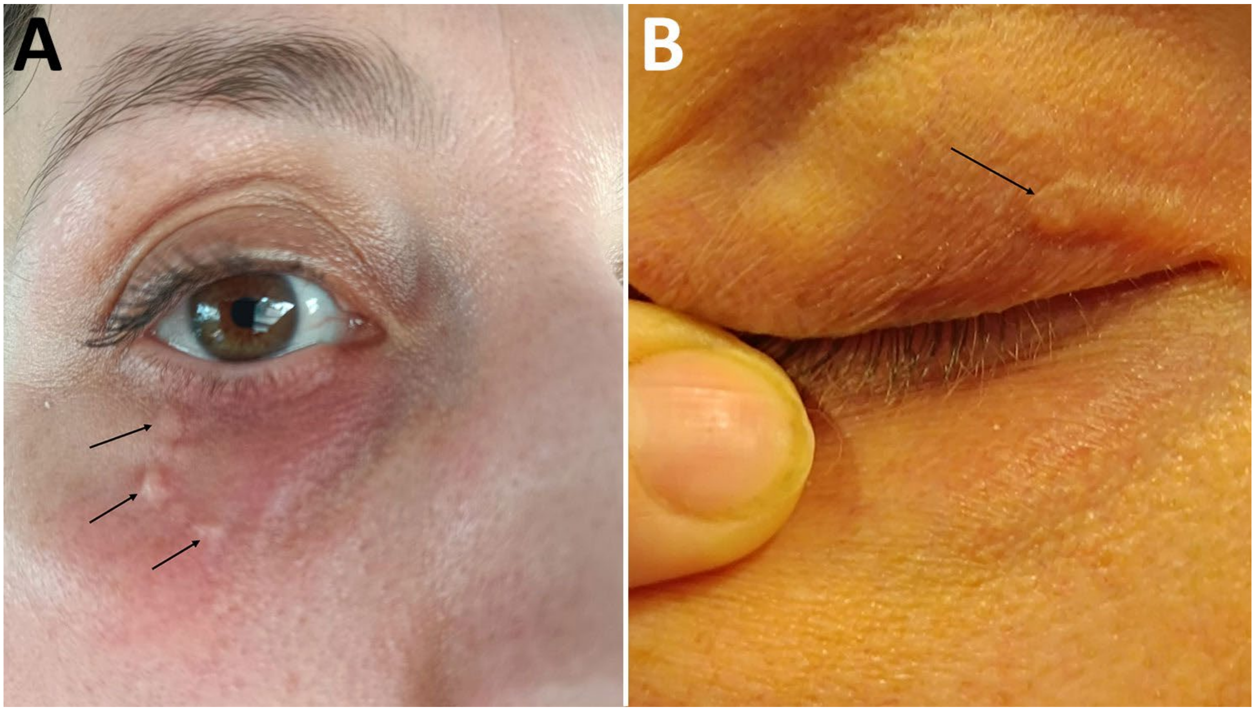

Appendix Figure. Macroscopic view of subcutaneous lesions caused by Dirofilaria repens, first in the suborbital region (A) and then migrated to the eyelid (B).
